# Charting the Future: Advanced Technologies for Sustainable Parasite Control in Aquaculture

**DOI:** 10.3390/ijms262110738

**Published:** 2025-11-04

**Authors:** Jiao Yang, Subha Bhassu, Arutchelvan Rajamanikam

**Affiliations:** 1 Animal Genetics and Genome Evolutionary Laboratory (AGAGEL), Department of Genetics and Microbiology, Institute of Biological Sciences, Faculty of Science, University of Malaya, Kuala Lumpur 50603, Malaysia; 22088751@siswa.um.edu.my; 2 Department of Parasitology, Faculty of Medicine, University of Malaya, Kuala Lumpur 50603, Malaysia

**Keywords:** aquaculture, parasite control, therapeutic method, sustainable technologies

## Abstract

Parasite control in aquaculture faces challenges primarily due to the drug resistance of traditional chemical treatments, as well as environmental pollution and toxicity. Aquaculture is among the fastest-growing food-producing sectors worldwide, yet parasite infections remain a significant challenge to productivity and sustainability. Emerging methods such as natural products, gene editing, immunotherapy, and auxiliary technologies like nanotechnology and biosensors are becoming alternative strategies for sustainable parasite control. These methods show significant potential, particularly in preventing drug resistance and reducing environmental impact. However, these approaches remain at an early research stage, with issues such as unstable efficacy, limited validation in field conditions and uncertain long-term safety hindering their translation into practice. This review synthesizes current advances, highlights these knowledge and application gaps, and outlines future directions for developing more reliable and sustainable parasite management strategies in aquaculture.

## 1. Introduction

According to data provided by Food and Agriculture Organization (FAO, 2024) [1], aquatic animal foods are an important source of high-quality protein, providing about 15% of the world population’s intake of animal proteins in 2021 and provided about 3.3 billion people with at least 20% of their average per capita intake of animal proteins. In 2022, global aquaculture production reached 130.9 million tonnes (≈USD 313 billion) and accounted for around 59% of total fisheries and aquaculture output. Focusing on aquatic animals, farmed production represented about 51% of the total (94.4 of 185.4 million tonnes) and supplied over 57% of aquatic animal foods for direct human consumption. This highlights the importance of aquatic foods as a key part of the global diet. However, parasite infections represent a significant issue in aquaculture, posing risks not only to food safety but also to the economic stability of industry. Many widely consumed fish are known hosts for certain parasites. For example, *Bothriocephalus acheilognathi* in grass carp (*Ctenopharyngodon idella*) can cause abdominal pain and hinder nutrient absorption in humans [2,3,4], while *Dibothriocephalus dendriticum* and *Dibothriocephalus latus* in trout and perch may lead to symptoms such as nausea, abdominal pain, vitamin B12 deficiency, diarrhea, and weight loss [5,6,7,8,9,10,11]. Globally, diseases caused by *Diphyllobothriidea* affect as many as 20 million people [7]. These fish-borne parasitic infections represent a significant food safety concern and are considered one of the major global foodborne zoonoses, with particularly high prevalence in some regions [12,13,14]. In aquaculture systems, several parasites have been documented to cause extremely high mortality and cost increases. For instance, *Oreochromis niloticus* in Upper Egypt showed an overall parasite prevalence of about 82% [15], while outbreaks of *Ichthyophthirius multifiliis* in freshwater aquaculture can cause severe mortality [16], with one study in India reporting up to 90% mortality in an affected farm [17]. In terms of economic impact, in marine systems, infestations of sea lice (*Lepeophtheirus salmonis*) impose a substantial economic burden on salmon aquaculture, with global control and production losses estimated at around £700 million annually and approximately £65 million per year in Scotland alone [18]. Farm-level assessments further indicate that sea lice management accounts for about 9% of the total farm-gate value in major producing countries [19]. Global annual losses from parasitic infections in farmed fish are estimated to range from approximately US$1.05 billion to US$9.58 billion, according to a report [20]. This represents roughly 0.3–3% of the total annual value of global aquaculture production, which may appear modest, yet it still translates into losses of hundreds of millions to nearly ten billion USD each year and should therefore be regarded as a significant concern for sustainable aquaculture development. And more recent evaluations indicate that the economic burden continues to rise, driven by increasing treatment costs and expanding parasite distribution in major aquaculture sectors [21,22,23].

In aquaculture, chemical treatments remain a primary approach for parasite control. For example, emamectin benzoate is used to treat *L. salmonis* infections and can reduce parasite loads by about 35% [24], hydrogen peroxide baths achieve approximately 74% delousing efficiency [24], and praziquantel shows nearly 100% therapeutic efficacy against fish tapeworms [25], and its removal rate for monogeneans often exceeds 80% [26]. These drugs can effectively reduce parasite loads in the short term, but long-term and repeated use has led to increased drug resistance and posed risks to the environment and food safety [27,28]. Residues of emamectin benzoate and fluazuron have been repeatedly detected in sediments and waters near Norwegian fish farms, with concentrations occasionally exceeding environmental quality standards [29]. Emamectin benzoate can persist in sediments for approximately 5–6 months (with a half-life of about 150–400 days), while fluazuron and teflubenzuron can still be detected 8–22 months after treatment cessation [29]. These chemicals can inhibit chitin synthesis in non-target crustaceans, causing molting disorders and developmental deformities in organisms such as shrimp and crabs, thereby posing long-term ecological risks [29]. In addition, chemical residues may also enter the food chain. A survey of farmed fish in China revealed that approximately 24–40% of edible fish samples from 19 provinces contained detectable antibiotic residues, reflecting the widespread use of chemical drugs and raising concerns about food safety [30]. Rising drug resistance and environmental concerns have driven a shift in *L. salmonis* control within the Norwegian salmon industry from chemical to mainly non-chemical methods in 2016 and 2017 [31]. Among these approaches, thermal and mechanical treatments can remove up to 70–80% of *L. salmonis*, but because they rely on temperature stimulation, brushing, and water jets, suboptimal operating conditions often lead to stress responses and skin lesions in salmon, with mortality rates of about 25–31% [31,32]. In contrast, freshwater and low-salinity baths are relatively mild osmotic treatments [33]. They are effective against common external parasites of marine fish, including *L. salmonis* [31], *Neoparamoeba perurans* [34], and *Neobenedenia* [35]. For Norwegian salmon, these treatments can remove 82–100% and about 95% of *L. salmonis*, respectively, although they are labor-intensive, consume large amounts of water, and may cause temporary osmotic stress [31]. These fish graze on visible, relatively large ectoparasites such as *L. salmonis* [36] and certain monogeneans [37], thereby helping to reduce parasite burdens. However, their mortality rates are high and are greatly influenced by temperature and rearing conditions. Overall, these non-chemical treatments help mitigate drug resistance and environmental contamination, but their applicability remains limited and, compared with chemical therapies, they may have more pronounced negative effects on fish growth and welfare [32,38].Given the limitations of chemical treatments, it is essential to explore alternative approaches for effective and sustainable parasite control in aquaculture. Promising alternatives include the use of natural plant extracts, which have shown potential for therapeutic applications with minimal environmental impact [39,40,41]. Predatory fungi offer another approach, targeting intestinal parasites within hosts without introducing synthetic chemicals into the ecosystem [42]. Advances in genetic technologies, particularly CRISPR, provide opportunities to enhance the innate resistance of aquaculture species against parasitic infections [43,44]. Furthermore, integrating these treatments with sensitive biosensors could enable real-time monitoring and timely interventions, optimizing control strategies [45].

This review summarizes current methods for parasite control and examines their potential applications in aquaculture, aiming to provide valuable insights that enhance food safety and sustainability in the aquaculture industry.

## 2. Natural-Based Approaches

### 2.1. Plant-Based Treatments

The use of herbal remedies in disease treatment has received increasing attention, particularly as an alternative approach to address microbial and parasitic infections, contributing to sustainable aquaculture practices [46,47]. Most of the plant extracts and secondary metabolites such as alkaloids, terpenoids and phenolic compounds are potent bioactive substances that can be used as antibiotics for disease treatment [40,48]. Herbal compounds, unlike chemical drugs, are typically mixtures of multiple active components. They often inhibit the physiological processes of parasites through synergistic effects, making it challenging for parasites to develop resistance to multiple compounds simultaneously and reducing the likelihood of resistance development [49]. Meanwhile, medicinal plants are more environmentally friendly, have lower side-effect profiles, are biodegradable, and do not accumulate as residues in animal tissues or the environment [50,51,52,53,54]. Moreover, plant-based medicines are often more cost-effective than chemical drugs, readily cultivable and accessible, making them extensively utilized in underdeveloped countries [47,55,56].

Many plant-based therapies have been proven effective for controlling parasites in aquaculture and can be administered through oral delivery or bath treatments [49]. Table 1 summarizes examples of treatments using natural drugs, including their active compounds, target parasites, effective concentrations, and therapeutic effects. These examples highlight the diversity and potential of herbal remedies in controlling aquaculture parasites. Table 1 shows that essential oils (EOs) are highly effective in killing *Euclinostomum heterostomum*. However, the authors noted that plant-derived EOs contain unstable compounds, which can potentially cause undesirable side effects in fish. They emphasized the importance of carefully distinguishing harmful compounds from beneficial ones during the refining process to achieve better results [57]. The treatment of *Gyrodactylus* spp. with saponins extracted from *Dioscorea collettii* var. *hypoglauca* (Table 1) was found to be effective only for internal parasites, showing no efficacy against external ones [58]. In laboratory studies, *Allium sativum* and *Terminalia catappa* were also highly effective in treating *Trichodina* sp. (Table 1). However, their effects were later found to be short-term, as the parasites reappeared after two weeks. Further research is needed to explore their potential for long-term control [59].

Moreover, the types and levels of bioactive substances in plants vary with their environment or health, resulting in differences in therapeutic effects [40]. Additionally, the low yield of active compounds in plants makes some natural drugs difficult to apply directly in aquaculture [60]. Although some studies suggest that herbal remedies have low toxicity to fish, research is still limited. Fish tolerance to these treatments also varies with age, species, and health status, making further studies essential to ensure their safe application in different fish species [49,61,62]. Herbal remedies are safer and more reliable than chemical pesticides. They have gradually replaced synthetic drugs and hormones as feed additives, but there is still significant potential for improvement [39]. To further their promising application in aquaculture, future research should prioritize long-term control mechanisms, toxicity evaluation, and production optimization, aiming to advance their widespread adoption in global sustainable aquaculture. And given the specificity of herbal treatments against parasites, efforts should also be directed toward the most common parasites in aquaculture and first address the most urgent challenges. Additionally, exploring common and easily accessible plants as herbal sources could help reduce costs and support the sustainable development of aquaculture.

**Table 1:** Herbal Treatment of Aquaculture Parasites.

Parasites	Fish Species	Drugs	Usage	Effect	References
*Euclinostomum heterostomum*	*Tilapia zillii*	*Verbesina alternifolia* EOs	24 h/600 mg/L	100% anthelmintic	[63]
		*Mentha piperita* EOs	24 h/1000 mg/L	50% anthelmintic	
*Dactylogyrus* spp.	*Poecilia reticulata* *Oreochromis niloticus*	*Lippia origanoides* EOs	5 min/100 mg/L	100% anthelmintic	[63]
		*Lippia sidoides* EOs	5 min/100 mg/L	100% anthelmintic	
*Gyrodactylus* spp.	*Poecilia reticulata* *Oreochromis niloticus*	Methanol extract of *Dioscorea collettii* var. *hypoglauca*	10 mg/L	100% anthelmintic	[58]
		Dioscin isolated from *D. collettii* var. *hypoglauca*	2 h/0.6 mg/L	100% anthelmintic (in vivo)	
*Neoechinorhynchus buttnerae*	*Tambaqui Colossoma macropomum*	Oleoresins from *Copaifera duckei*	24 h/0.1868 mg/mL	100% anthelmintic	[62]
		Oleoresins from *Copaifera pubiflora*	24 h/0.1868 mg/mL	100% anthelmintic	
		Oleoresins from *Copaifera reticulata*	24 h/0.1868 mg/mL	100% anthelmintic	
*Neobenedenia* *girellae*	marine fishes (in vitro assay)	Pomegranate extract	8 h/62.5 mg/L	100% anthelmintic	[64]
*Trichodina* sp.	*Oreochromis niloticus*	*Allium sativum*	2 d/800 ppm	100% anthelmintic	[59]
		*Terminalia catappa*	2 d/800 ppm	100% anthelmintic	
*Gyrodactylus*	*Oreochromis niloticus*	crushed garlic cloves (*Allium sativum*)	300 mg/L	68% elimination of disease	[65]
		garlic oil (*Allium sativum*)	4 h/2, 2.5 and 3 ppt	100% anthelmintic	
*Gyrodactyluskobayashii*	*Carassius auratus* (model fish)	plant-derived plumbagin	30–60 min/0.4–0.7 mg/L	100% anthelmintic	[60]

### 2.2. Microbial-Based Approaches

#### Probiotics

Probiotics are live microorganisms that provide health benefits and are widely used in animal production and human health protection [66,67,68]. Many probiotic products have been commercially used in aquaculture as feed additives and alternatives to antibiotics [68,69,70]. In aquaculture, where farmed animals closely interact with the microbial communities of their surrounding water, the concept of probiotics has been broadened and is sometimes described as microbial “water additives” [71]. Verschuere et al. [72] defined aquaculture probiotics as “live microbial adjuncts which have a beneficial effect on the host by modifying the host-associated or ambient microbial community, by ensuring improved use of the feed or enhancing its nutritional value, by enhancing the host response towards diseases, or by improving the quality of its environment.” In aquaculture applications, inactivated microorganisms or their cellular components have also been used as additives with probiotic-like functions to improve host health or the microbial balance of the rearing environment [73]. These preparations are now more accurately referred to as parabiotics and postbiotics. Parabiotics describe non-viable or inactivated microbial cells that still retain functional activity, whereas postbiotics refer to cell-free metabolites, secretions, or other microbial by-products that exert beneficial effects on the host [74]. Because both parabiotics and postbiotics are essentially composed of molecules rather than live organisms, they can successfully withstand adverse digestive and physiological conditions and may serve as suitable alternatives to traditional probiotics in aquaculture applications [74].

Probiotics include various types of bacteria, bacteriophages, microalgae, and yeasts [75], which can enhance fish resistance to pathogens, protect their health, or promote growth [76]. The use of probiotics to combat bacterial and viral infections in aquaculture is currently the most extensively studied [77,78]. Extensive research has proven that fish treated with probiotics have higher survival rates after pathogen exposure [79,80,81]. Feed additives and direct addition to water are the two main approaches to delivering aquatic probiotics [82,83]. Their modes of action are multifactorial, involving mechanisms such as competitive exclusion, metabolite inhibition, and immune modulation. Probiotics compete with pathogens for attachment sites and nutrients, preventing their colonization in the host. They also secrete metabolites such as antimicrobial peptides, lactic acid, and bacteriocins, which directly inhibit pathogen growth. Furthermore, probiotics enhance the resistance of the host to infections by stimulating the innate immune system through the activation of inflammatory cytokines or by boosting macrophage activity [76,77,84,85,86].

The use of probiotics in treating aquaculture parasites is less common, as they are less effective against ectoparasites and endoparasites compared to their use in treating bacterial pathogens [69,77,87]. The mechanisms by which probiotics treat parasitic infections remain largely unknown and may be similar to those used in resisting bacterial infections [77,87]. However, due to the size differences between parasites and microorganisms, competition for nutrients may only serve as a mechanism against smaller parasitic pathogens, such as *Saprolegnia* sp. [77,88]. In addition, probiotics indirectly reduce the risk of parasite transmission by regulating microbial communities in the water. This effect may involve depleting nutrients in the water, degrading pollutants, and adjusting pH levels to create an environment unfavorable for pathogen survival [77,82,83,89,90].

Although there is less research on treating parasitic infections with probiotics, it still holds great potential [77]. Nurhajati et al. demonstrated that *Lactobacillus plantarum* FNCC 226 has the ability to inhibit *Saprolegnia parasitica* A3 both in vivo and in vitro in catfish (*Pangasius hypophthalmus*) (Table 2) [88]. *Bacillus subtilis* not only serves as a probiotic additive to promote the growth of fish and shrimp, enhance immune responses, and improve disease resistance but also acts as an antigen delivery vehicle for parasite control (Table 2) [91,92,93]. Recombinant spores expressing *Clonorchis sinensis* paramyosin (CsPmy) (*B. subtilis*-CotC-CsPmy) were used to vaccinate grass carp (*Ctenopharyngodon idella*), significantly reducing the cercarial burden in the fish (Table 2) [85]. According to Pieters et al. [94], *Aeromonas sobria* GC2, at a concentration of 10^8^ cells per gram of feed, was highly effective in treating *Ichthyophthirius multifiliis* on fish skin, reducing mortality from 98% to 0% (Table 2). In contrast, another probiotic, *Brochotrix thermosphacta* BA211, showed no effect [94]. Effective Microorganisms (EMs), as a probiotic formulation, have shown significant improvement in managing *Trichodina* infections in fish (Table 2). However, their effects last only 15 days, and high concentrations of EMs may cause toxicity, reducing their protective efficacy [95]. Ornamental fish and farmed fish face similar parasitic issues, such as common gill parasites (e.g., *Myxobolus* sp.) or skin parasites [96]. In a study on *Cyprinus carpio* infected with *Myxobolus* sp., a probiotic solution at a concentration of 0.55 mL/30 L effectively reduced gill tissue damage caused by the infection (Table 2). This approach could be extended to food fish suffering from similar infections [96].

The use of microbial probiotics for health maintenance, disease prevention, and control is now widely recognized as a new eco-friendly alternative for sustainable aquaculture [97]. However, the widespread commercialization of probiotics for parasite control in aquaculture still has a long way to go. The selection process for suitable probiotics is challenging, as qualified probiotics must lack plasmid-encoded antibiotic resistance genes, tolerate a wide range of pH levels, and pose no pathogenic risk to humans or animals [76,84]. It is also unclear how long probiotics can survive in the host and whether they can establish long-term colonization to provide sustained health benefits, requiring further research [98]. In many cases, improper probiotic supplementation can also lead to negative effects on fish growth, health and disease resistance [99,100]. Additionally, probiotic products currently sold in some countries are poorly regulated, often lacking specific labeling of species or strains, and suffer from serious quality issues [101]. Despite these challenges, probiotics remain a promising and environmentally friendly approach for parasite management in aquaculture. However, due to the larger size and complex life cycle of parasites, probiotic mechanisms that effectively target bacterial pathogens may not function in the same way against parasites. Further research is needed to elucidate these interactions, which could help refine treatment strategies and address challenges such as short-lived efficacy and potential toxicity at high concentrations. A deeper understanding of these mechanisms may also facilitate the development of more targeted and effective probiotic-based treatments. With continued research and development, overcoming these limitations could lead to more reliable and sustainable probiotic applications in aquaculture.

**Table 2:** Probiotic Applications for Parasite Control in Aquaculture.

Parasites	Fish Species	Probiotic	Probiotic Administration Route	References
*Saprolegnia parasitica* A3	*Pangasius hypophthalamus Sauvage*	*Lactobacillus plantarum* FNCC 226	Immersion	[88]
*Clonorchis sinensis*	*Ctenopharyngodon idella*	*Bacillus subtilis*	Diet	[85]
*Ichthyophthirius multifiliis*	*Oncorhynchus mykiss*	*Aeromonas sobria* GC2	Diet	[94]
*Trichodina*	*Oreochromis niloticus*	Effective Microorganisms (EMs)	Diet	[95]
*Myxobolus* sp.	*Cyprinus carpio*	*Bacillus* spp., *Lactobacillus* sp. and *Nitrosomonas* sp.	Immersion	[96]

## 3. Genetic and Molecular Therapeutic Approaches

Gene editing technology is a powerful tool for precise manipulation of the genome. Through gene knock-in, knock-out, or modulation of gene expression, we are able to modify specific genes in organisms to improve disease resistance, such as strengthening resistance genes in the host or silencing functional genes associated with pathogens [102].

### 3.1. RNA Interference (RNAi)

RNA interference (RNAi) is an innate immune mechanism based on double-stranded RNA (dsRNA) within cells [103,104,105]. It was first discovered by Fire et al. (1998), who demonstrated in *Caenorhabditis elegans* that dsRNA could specifically inhibit gene expression [106]. RNA interference (RNAi) initiates intracellular gene silencing pathways by introducing exogenous or endogenous double-stranded RNA (dsRNA). This technology has been extensively studied in aquaculture. It is considered a promising tool to combat diseases caused by viruses, parasites, and bacteria [105]. RNAi-induced gene silencing is transient, usually suppressing specific gene expression for a limited duration. Exogenous dsRNA is typically degraded or cleared from organisms within a few days to weeks. This reversible nature reduces long-term impacts on both the environment and organisms, making RNAi a promising and safe tool for gene editing [105,107,108,109].

*Neobenedenia girellae* is a significant parasite that poses a severe threat to marine aquaculture species such as amberjack (*Seriola dumerili*) and yellowtail (*Seriola quinqueradiata*). Currently, there are no effective control methods available. Ohashi et al. [110] successfully silenced two key genes, *Ngvlg1* and *Ngvlg2*, associated with germ cell development, by immersing *Neobenedenia girellae* in a solution containing dsRNA. This resulted in a substantial reduction in the quantity and quality of germ cells in the parasite, significantly lowering egg hatchability. These findings offer promising prospects for developing sterilization-based methods to control parasitic infections [110]. In a study on the parasite *Heterosporis saurida* infecting lizardfish (*Saurida undosquamis*), Saleh et al. [111] used RNAi technology to silence two key genes—ATP/ADP antiporter 1 and methionine aminopeptidase II. This significantly reduced the number of spores and decreased initial infection levels by 40% and 60%, respectively. Combined silencing of both genes showed an even greater effect [111]. Salmon is an economically important fish species, and *Myxobolus cerebralis* is known to cause whirling disease in salmon. Sarker et al. [112] successfully disrupted the parasite’s lifecycle by silencing the *MyxSP-1* gene in its intermediate host, *Tubifex tubifex*. Fish fry exposed to treated *T. tubifex* showed no signs of infection [112]. *Lepeophtheirus salmonis* is another significant parasite that affects salmon. Silencing its molting-associated chitinase gene (*LsChi2*) resulted in abnormal swimming behavior and functional impairments. This demonstrated significant gene regulation effects and highlighted the potential for developing novel therapeutic approaches against *L. salmonis* [113].

However, the application of RNAi in aquaculture still faces several challenges. In the study by Saleh et al. [111], the knockdown effects on parasite-related genes began to diminish after seven days, indicating the transient nature of RNAi suppression. Further optimization is needed to enhance its durability and practical application potential [111]. Eichner et al. [113] successfully impaired the locomotive function of parasites using RNAi technology. However, the effect was limited to specific developmental stages of the parasite and could not target the entire lifecycle [113]. Specificity and the potential for off-target effects are also challenges for RNAi. Designed dsRNA or siRNA may not perfectly complement the target gene, potentially binding to non-target mRNA and causing unintended gene silencing. This off-target effect, primarily linked to immune issues, can lead to cytotoxicity, disrupt normal cellular functions, and result in metabolic disorders or cell death [105,114]. Introduced dsRNA may be recognized by the immune system as a foreign molecule, triggering unintended inflammatory responses or cytotoxicity, which can harm host health [105,109]. Additionally, naked molecules face difficulties in penetrating cell membranes and are easily degraded by nucleases, making it essential to find effective delivery vectors [109]. Although dsRNA remains in the environment for a limited time, the application of this treatment through bathing or feeding still poses a risk of affecting non-target species in open-water systems. To enable the large-scale application of RNAi in aquaculture, future research should focus on enhancing the stability of dsRNA in the environment, reducing off-target effects, and exploring efficient delivery methods.

### 3.2. CRISPR/Cas9

CRISPR is a promising gene editing tool to modify disease-resistant genes in aquatic populations. It potentially reduces pathogen numbers and transmission rates, thereby improving the health of aquatic animals [115]. However, its research and application in aquaculture disease control are relatively limited compared to RNAi technology. There are few case studies for controlling parasitic infections.

This genetic modification is permanent, as it directly alters the organism’s genome, potentially resulting in the transmission of the modified genes to future generations [115]. If fish with altered genes escape into the natural environment, they could potentially impact the ecosystem [116]. Gene modification may bring fish specific advantages or disadvantages. For example, fish with enhanced growth hormone-related genes may gain a reproductive advantage in the wild due to their larger size and earlier maturation. However, their hybrid offspring have reduced adaptability to the wild, which results in higher mortality rates during the juvenile stage, leading to a gradual decrease in wild fish populations and disrupting the ecological balance [117,118,119]. This risk can be minimized by introducing sterility genes, which have been successfully tested in Atlantic salmon (*Salmo salar*) in Norway [120,121].

The acceptance of gene editing technology by the public is an important consideration as concerns about the safety of genetically modified foods prevails. This fast and targeted approach is quite different from the slower, more familiar process of selective breeding [115]. In the future, concerns regarding safety, environmental impact, and other issues need to be addressed through transparent and scientific evidence, along with more effective implementation strategies, to communicate with the public.

## 4. Immune-Based Approaches

### 4.1. Vaccine

Vaccination is an eco-friendly and sustainable disease control method with great potential for development in the aquaculture industry [122]. Vaccines can be classified into live, inactivated, and genetically engineered according to its preparation method [123]. Live vaccines use attenuated pathogens to mimic the natural infection process and induce an immune response. Inactivated vaccines use physical or chemical methods to inactivate pathogens while retaining their immunogenicity to stimulate the host’s immune system. Genetically engineered vaccines, including recombinant subunit vaccines, nucleic acid vaccines, protein-engineered vaccines, and others, are designed using modern biotechnologies to express pathogen-specific antigens and trigger protective immune responses [124]. Although live vaccines offer the best protection, they carry the risk of re-establishing virulence. Genetically engineered vaccines are costly to develop and generally provide more limited protection. As a result, in aquaculture, commercially available vaccines are primarily inactivated vaccines, which are safe and effective [124,125].

Currently, most research on aquaculture vaccines focuses on bacterial diseases, with fewer studies on parasitic infections [126]. Taking South Korea as an example, among the 29 commercially approved vaccines by the end of 2019, only 2 can prevent parasitic diseases [127]. However, existing research has provided valuable insights for the development of parasitic vaccines. *Cryptocaryon irritans*, a major parasite threatening marine aquaculture fish, can cause massive fish mortality and result in significant economic losses [128,129]. Josepriya et al. [129] developed a DNA vaccine combining the heat shock protein 70C (Hsp70C) adjuvant with immobilized antigen (iAg). Experiments showed that the vaccine significantly enhanced the immune response in fish, achieving a 100% relative protection rate after infection. The protective effect gradually declined after 1.5 months, but this study provides an important reference for parasitic vaccine development [129]. In another study, researchers showed that inactivated *Cryptocaryon irritans* vaccines significantly improved parasite resistance in orange-spotted grouper (*Epinephelus coioides*) by enhancing both specific and non-specific immune responses. In parasite challenge experiments, the survival rate in the high-dose inactivated vaccine group reached 80%, further confirming the critical role of vaccine dosage in immune protection [130]. *Miamiensis avidus* is also a harmful parasite to marine fish. Research has shown that inactivated vaccines encapsulated in chitosan microspheres (chitosan MS) provide up to 57.1% relative protection (RPS) in olive flounder (*Paralichthys olivaceus*) through oral immunization, significantly increasing the antibody levels against *M. avidus* in the fish [131,132].

Fish vaccines are safe for the ecosystem and have minimal side effects on the organisms [124,133]. However, there are still many challenges in commercial application. A major limitation in parasitic vaccine development is the difficulty in obtaining large quantities of high-quality parasites for antigen production. Many fish parasites have complex, poorly understood life cycles, making in vitro culturing a significant challenge [134]. This limitation constrains not only live and inactivated vaccine development but also antigen characterization and immune response studies. Although genetically engineered vaccines can be developed as alternatives, their protective efficacy is weaker compared to traditional vaccines. Additionally, there are currently no reports of successful polyvalent vaccines for fish parasites [20]. There is also insufficient research on host–parasite interactions and immune responses, which presents a challenge for vaccine development [20].

Next, administration of fish vaccination is also facing difficulties. Injection, bathing, and oral are the three common methods of vaccine delivery in aquaculture [135]. Injection is the most effective method, but it is labor-intensive and not the best approach for large-scale population vaccination in practical production [136,137]. Oral administration is considered the most feasible form of immunization for aquatic species and can be applied to various types of vaccines. However, vaccines are susceptible to degradation by enzymes in the digestive tract, so it is necessary to avoid delivering naked antigens. Encapsulating materials such as alginate, chitosan, liposomes, and other polymeric microspheres or biofilms should be explored. Alternatively, simpler and more cost-effective biological carriers, such as insects, probiotics, and plants, may be used [138,139,140,141]. Moreover, the number of fish receiving oral immunization and the vaccine dose are variable, making the outcome unpredictable. In some cases, the effectiveness is only half that of injectable vaccines [124]. Immersion is a unique vaccination method for aquatic species that can quickly and effectively activate a systemic immune response with relatively low labor intensity. However, to achieve optimal immune effects, it may be necessary to increase the dosage, add adjuvants, or administer multiple doses [142,143]. Finally, insufficient funding for vaccine development, along with the diverse approval processes and requirements, are major barriers to the commercialization of vaccines [126,144]. At present, many aquaculture vaccines are still in the clinical trial stage. However, with ongoing advancements in gene sequencing, antigen screening, and mucosal immunity research, along with the development of novel antigen expression and delivery technologies, aquaculture vaccines are expected to make significant breakthroughs in the future, providing a major boost to the aquaculture industry [124,144]. Future studies should focus on optimizing antigen production, such as using plant-based or insect cell expression systems to improve scalability and cost-effectiveness. Additionally, novel delivery approaches, including nanoparticle-based encapsulation and probiotic-mediated antigen transport, may enhance vaccine stability and efficacy. Addressing these challenges through interdisciplinary research could accelerate the development of effective parasitic vaccines and facilitate their large-scale application in aquaculture.

### 4.2. Immunostimulant

Immunostimulants are also a promising new treatment method. As an eco-friendly and safe strategy for parasite control, they have gradually become a hotspot for research and application. Immunostimulants work by activating the nonspecific immune system in fish, enhancing their overall immunity, and thus indirectly reducing parasite infections [145]. Moreover, for juvenile fish that are unsuitable for vaccination, immunostimulants can serve as a good alternative [145].

β-Glucans are one of the most widely studied immunostimulants in aquaculture, primarily derived from organisms such as yeast, fungi, and algae [145,146]. It can bind to receptors on the surface of various immune cells, triggering an immune response [147,148], and enhance the host’s resistance to pathogens such as parasites, fungi, and bacteria [149,150]. Microsporidia are widely distributed in marine, freshwater, and estuarine areas, posing a significant threat to aquaculture [151]. Research by Guselle et al. [152] demonstrated that after intraperitoneal injection of glucan in rainbow trout (*Oncorhynchus mykiss*), the number of *Loma salmonae* (Microsporidia) decreased by 90%, showing the potential of glucan as an immunostimulant in parasite control. Furthermore, it was found that the immune effect of glucan is time-dependent. Administering the treatment 21 days before parasite exposure produced the best results, significantly reducing gill hyperplasia, whereas treatment after infection proved to be less effective. The effect of β-glucan is highly dependent on the timing of administration [152]. In a study on *Mesanophrys* sp. infection in swimming crabs (*Portunus trituberculatus*), it was found that β-1,3-glucan, as an immunostimulant, significantly increased the enzymatic activity of hemocytes, including phenoloxidase, lysozyme, and superoxide dismutase (SOD). By regulating the expression of immune-related genes, it effectively inhibited the growth of the parasite *Mesanophrys* sp. and exhibited a dose-dependent immune protection effect [153].

Microalgae have recently been recognized as sustainable sources of immunostimulants in aquaculture. Representative genera include *Spirulina*, *Chlorella*, *Haematococcus*, and *Porphyridium*, which provide a range of bioactive compounds such as sulfated polysaccharides (SPs), polyunsaturated fatty acids (EPA and DHA), carotenoids (astaxanthin), and phycobiliproteins (phycocyanin) [154,155,156]. Among these compounds, polysaccharides enhance macrophage phagocytic activity and promote the expression of pro-inflammatory cytokine genes, activating the innate immune response [157]. Astaxanthin improves stress tolerance and immune performance in fish. Acting as a strong antioxidant, it scavenges reactive oxygen species and regulates redox-sensitive signaling pathways, helping to maintain tissue homeostasis and overall fish health [158,159]. In shrimp, a 30-day supplementation with 1.0% (*w*/*w*) sulfated polysaccharides from *Porphyridium cruentum* increased total hemocyte count and phenoloxidase activity in *Litopenaeus vannamei*. Survival after *Vibrio* challenge reached nearly 90%, reflecting stronger nonspecific defense and resistance to infection [160]. Feeding *L. vannamei* a diet containing 1% *Chlorella vulgaris* hot water extract (CVE) also enhanced enzyme activity, improved antioxidant status, and increased tolerance to ammonia stress, indicating protection under stress–infection conditions [161]. For fish, *Spirulina platensis* at 10 g/kg feed improved feed conversion efficiency in Nile tilapia (*Oreochromis niloticus*) and provided 22.2% relative protection against *Edwardsiella tarda* infection [162]. Astaxanthin from *Haematococcus pluvialis* at 25–75 mg/kg feed strengthened the intestinal barrier and boosted antioxidant and immune status in rainbow trout (*Oncorhynchus mykiss*), whereas higher doses inhibited growth, highlighting the need for an optimal dosage range [163]. Most aquaculture studies examining immunostimulants and pathogen resistance have focused on bacterial or viral infections, and parasite challenge evidence remains limited. Still, marine-derived sulfated polysaccharides have shown clear inhibitory or antagonistic effects against a range of marine parasites, combined with low toxicity and immunomodulatory activity. These findings support the potential use of algal polysaccharides in feed to enhance resistance to parasitic infections [164].

In addition, animal-derived substances such as chitosan and lactoferrin, and plant-derived compounds such as polyphenols, flavonoids, and saponins mentioned before, can also serve as immunostimulants. Under certain conditions, they stimulate the host’s immune system, thereby enhancing fish resistance to pathogens [146]. However, their practical application presents several challenges. Careful selection of immunostimulants and precise dosage control are essential to achieving optimal results, as excessive use may lead to immunosuppression and other adverse effects [165]. Although pre-infection administration of immunostimulants offers optimal protection, the unpredictable nature of disease outbreaks in aquaculture makes precise timing difficult. Additionally, individual variations in immune responses complicate dosage standardization [166]. Given the potential risks of immunosuppression and immune dysregulation, long-term use is not a viable strategy [167,168]. Therefore, immunostimulants should be considered a short-term immune-enhancing strategy rather than a long-term solution. Future research should focus on developing predictive disease monitoring systems to optimize their application by integrating biomarker-based immune assessments with environmental risk factors. Additionally, combining immunostimulants with complementary disease control methods, such as vaccination or functional feeds, may enhance their effectiveness and reduce the need for continuous administration.

Table 3 provides a comparative summary of the advantages and disadvantages of key parasite control techniques in aquaculture.

## 5. Environmental Control Strategies

**Figure 1:**
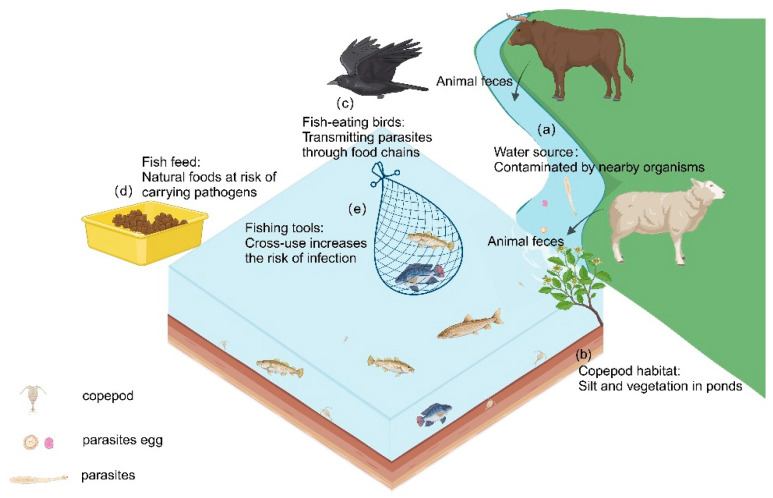
Potential influences on parasitic infections in aquaculture systems. Inadequate selection of water sources for fisheries and the presence of contamination (**a**). Clearing pond silt and vegetation before culturing to reduce copepods, one of the intermediate hosts of the parasite (**b**). Avoid establishing fisheries where fish-eating birds are present to prevent the transfer of parasites between fishponds (**c**). Use pelleted fish feed instead of natural foods such as small fish and shrimp (**d**). Ensure clean equipment for mid-term fishpond maintenance and harvesting (**e**). (Created with BioRender.com).

Parasites mainly enter aquaculture systems through the environment. Common sources include contaminated water, fish and birds carrying pathogens, and pollutants such as animal feces (Figure 1) [169]. These sources of pollution spread rapidly through the ecological chain, allowing parasites to propagate within aquaculture systems and increasing management complexity. Compared to post-infection treatments, optimizing the environment to interrupt transmission pathways is a more effective approach. Environmental management not only reduces infection risks at the source but also decreases dependence on drugs, helping to avoid resistance issues and environmental pollution. Therefore, implementing scientific environmental management measures at different stages of aquaculture can effectively block the transmission routes of parasites, thus reducing the risk of infection.

### 5.1. Water Source Management

The water source is the core of the aquaculture system, and its quality directly determines the risk of parasitic infections. Contaminated water often becomes a primary transmission route as it carries parasite eggs or larvae [170]. Therefore, when selecting a water supply, priority should be given to relatively clean sources. Filtration and sterilization systems, such as ozone treatment or ultraviolet (UV) sterilization, can be implemented to ensure safety [171,172], and studies have shown that these methods can effectively reduce *Cryptocaryon irritans* infections in aquaculture farms [173].

### 5.2. Pond Cleaning and Environmental Safety

The sediment and aquatic plants in ponds provide a suitable habitat for parasites and their intermediate hosts. Regular cleaning helps eliminate these habitats, reducing the number of parasites and their intermediate hosts [169,174,175]. Thorough pond drying and chemical disinfection before stocking fish fry can effectively reduce the presence of intermediate hosts, such as copepods and snails, thus lowering the overall risk of parasite transmission [176]. In addition, preventing environmental pollution around the farm is crucial, especially to limit the activity of pathogen carriers such as birds. Installing isolation nets or bird deterrents can effectively reduce the risk of parasites being transmitted from wildlife to the aquaculture system [169].

### 5.3. Density and Feeding Management

Properly regulating stocking density is an important measure for disease management. High stocking densities increase the frequency of fish interactions, promoting the spread of parasites. In a study on parasite infections in farmed koi (*Cyprinus carpio*), it was found that higher stocking densities were associated with higher rates of parasitic infections [177]. Moreover, high stocking densities can lead to excessive oxygen consumption and increased water pollution, further adding stress to the fish [171,178]. Reducing stocking density and providing a more comfortable growing environment can significantly lower the risk of parasitic infections, while also improving fish growth rates and overall health. Additionally, feed management is equally important. Avoiding natural feeds that may contain parasite eggs, such as small fish and shrimp, and prioritizing processed pellet feeds can effectively disrupt the food chain transmission of parasites.

### 5.4. Water Quality Maintenance

Stable water quality is a critical foundation for controlling parasitic infections. Pollutants in the water and fluctuations in water quality parameters, such as insufficient dissolved oxygen, high ammonia levels, or abnormal pH, can weaken fish immunity and create favorable conditions for parasite reproduction [179]. Therefore, aquaculture farmers should maintain water quality within a healthy range through appropriate water exchange frequency, oxygenation measures, and pollution control. Research by Pan et al. (2024) has shown that ponds with shorter water exchange intervals and more frequent water quality monitoring and adjustments tend to have lower parasitic disease prevalence [180]. Modern technologies, such as biosensors, can be used to regularly monitor water quality parameters, providing data support for timely adjustments in management strategies. These methods help reduce environmental stress, improve fish health, and further decrease the risk of parasite transmission.

### 5.5. Tool Management and Wastewater Treatment

During the entire aquaculture process, the cross-use of tools such as fishing nets and fish baskets poses a potential route for parasite transmission. If these tools are not properly cleaned and disinfected, they may carry parasite eggs or pathogens and spread them between different aquaculture ponds. Therefore, it is recommended to implement strict cleaning protocols for tools in daily management, using high-temperature treatments or appropriate chemical disinfectants to ensure complete disinfection before use, thereby interrupting transmission pathways.

In addition, aquaculture wastewater often contains fish excrement, undigested feed residues, and potentially parasite eggs. To ensure environmental safety and prevent further spread of parasites, basic sedimentation and filtration treatments should be carried out before discharging the wastewater. These measures not only help protect the surrounding ecosystem but also improve the overall environmental hygiene of the aquaculture farm.

**Figure 2:**
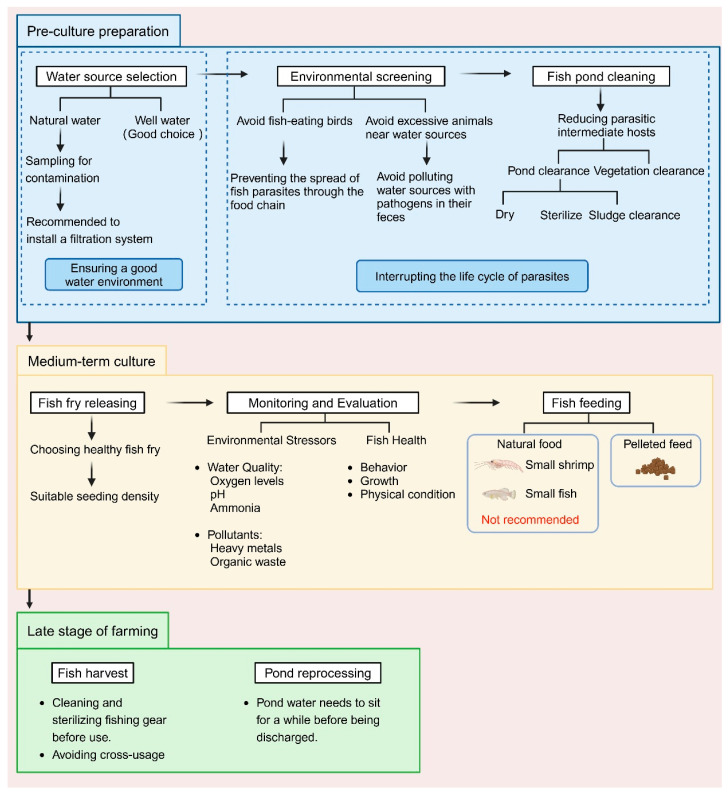
Parasite preventive measures during the process of aquaculture (Created with BioRender.com).

In conclusion, scientific environmental management is a key strategy for controlling parasitic infections and should be implemented throughout the entire aquaculture process. From the initial stages of water source selection and pond cleaning to scientific feeding, stocking density control, tool management, and water quality maintenance during the farming process, and finally to wastewater treatment at the end of the farming cycle, the collaborative efforts of each stage effectively interrupt the transmission pathways of parasites (Figure 2). These systematic measures enable aquaculture farms to reduce the risk of parasitic infections, minimize the use of medications, improve farming efficiency, and promote the sustainable development of aquaculture.

## 6. Application of Modern Auxiliary Technologies

In aquaculture, modern auxiliary technologies have provided innovative ways to improve parasite control and overall farm management. Among them, nanotechnology and biosensor-based systems address different aspects of disease prevention and control. Nanoparticles can enhance therapeutic efficacy by improving drug delivery or acting directly against parasites, while biosensors allow rapid and sensitive detection of pathogens for timely intervention. Together, these technologies strengthen disease surveillance and treatment capacity, contributing to more effective parasite management and the sustainable development of aquaculture.

### 6.1. Nanoparticles

Particles ranging from 1 to 100 nanometers in size are referred to as nanoparticles (NPs), which can be classified into various types, including Metal Nanoparticles (MNPs), Carbon-Based Nanoparticles (CBNs), Lipid Nanoparticles (LNPs), and Polymeric Nanoparticles (PNPs) [181,182]. Its small size allows it to easily cross various biological barriers, while its high surface-to-volume ratio provides more sites for interaction with compounds [183]. These characteristics enable nanotechnology to demonstrate wide application potential in various fields, including food, healthcare, agriculture, and industry [184,185]. One of the most widespread applications is as a delivery carrier for therapeutic molecules, such as drugs or vaccines [186]. As nanotechnology gains attention in various fields, its potential in aquaculture has also been increasingly recognized, demonstrating unique value in optimizing vaccine delivery and pathogen control.

In aquaculture drug delivery, nanoparticles have gradually become a focus of research due to their unique advantages in drug protection and delivery. They can protect drugs or vaccines during the delivery process, preventing degradation caused by environmental pH changes and gastrointestinal digestive enzymes, thereby ensuring the stability of active molecules [187,188]. In addition, certain nanoparticles also possess good biocompatibility and biodegradability, allowing them to break down into harmless products within the body and be eliminated through natural metabolic processes [189]. For nanoparticles with slower degradation rates, they can gradually release drugs or antigens, extending the duration of action and enhancing the immune response [190]. Based on these advantages, the application of nanoparticles in aquaculture shows great potential.

Among the various types of nanoparticles, chitosan nanoparticles (CNPs) and poly (lactic-co-glycolic acid) (PLGA) nanoparticles are the most widely studied carriers. They combine the advantages mentioned above and are extensively used in the control of bacterial and viral diseases in aquaculture [191,192]. PLGA nanoparticles have shown significant advantages in treating aquatic animal diseases (Table 4). CNPs and LNPs also perform excellently in vaccine delivery and gene editing applications (Table 4). By encapsulating double-stranded RNA (dsRNA), nanoparticles enable precise targeting and silencing of pathogen genes, thereby preventing disease transmission. In addition, RNAi drugs delivered by LNPs have been approved by the U.S. Food and Drug Administration (FDA), further demonstrating their potential for clinical application [193].

While nanoparticles are best known for their role as drug delivery carriers in aquaculture disease treatment, recent findings have revealed that certain nanoparticles can also be directly applied to treat parasitic infections. This direct therapeutic potential has attracted significant attention, demonstrating their potential as effective tools for pest and parasite management (Table 4) [194]. Silver nanoparticles (AgNPs) [195] are the most widely studied and show broad antiparasitic activity against protozoan, monogenean, and crustacean parasites (Table 4). Recent studies have also reported the successful use of other nanoparticles, such as chitosan and iron oxide formulations, against a variety of fish parasites (Table 4). Although the formulations and exposure conditions differ, these studies consistently report similar outcomes—rapid parasite mortality, inhibition of reproduction, or detachment from host tissues following nanoparticle exposure. Microscopic observations have revealed severe deformation and rupture of parasite surfaces, while molecular analyses in *Argulus* have shown the upregulation of ion-channel–related genes, suggesting neuromuscular disruption. Together, these findings suggest that nanoparticles may exert antiparasitic activity mainly through physical and oxidative damage to parasite surfaces and by interfering with ionic or metabolic homeostasis. These effects highlight their potential as eco-friendly agents for managing both ecto- and endoparasitic infections in aquaculture. Apart from their therapeutic effects, NPs also have significant applications in the rapid diagnosis of pathogens. For example, an electrochemical DNA sensor based on AuNPs can be used to detect the fish pathogen *Aphanomyces invadans* [189]. This technology offers higher sensitivity and faster detection compared to traditional methods, providing valuable time for the early detection and treatment of parasitic diseases [196]. This topic will be further discussed in the following section on biosensors.

Although NPs has made significant progress in aquaculture, most research has focused on bacterial and viral diseases, with less emphasis on parasitic disease control [197,198]. Existing studies have demonstrated that some NPs have direct insecticidal effects, but their specific mechanisms of action during the multi-stage lifecycle of parasites remain unclear [194]. In addition, the potential impact of NPs on aquatic organisms and the environment needs further evaluation. For example, studies have shown that certain metal NPs, due to their non-biodegradable nature, may induce inflammation, oxidative stress, and DNA damage by penetrating cell membranes [199,200,201]. At the same time, the bioaccumulation of NPs in fish tissues may pose a risk of transmission along the food chain, potentially leading to adverse effects on ecosystems and human health [170,202]. As treatment methods for parasites continue to develop, research on the application of NPs in parasite control is expected to increase. With their unique advantages in precise delivery and targeted therapy, NPs are a highly promising approach in the field of parasitic treatment.

**Table 4:** Applications of Nanoparticles (NPs) in Aquaculture.

Type of Nanoparticles (NPs)	Application	Key Findings	References
Poly (lactic-co-glycolic acid) (PLGA)	Encapsulation of rifampicin to treat *Mycobacterium marinum* infections in zebrafish embryos (Danio rerio)	More effective than rifampicin alone; significantly improves embryo survival rates	[203]
Chitosan nanoparticles (CNPs)	Delivery of dsRNA to control Yellow Head Virus (YHV) and White Spot Syndrome Virus (WSSV)	Precisely targets pathogen genes; significantly improves shrimp survival rates	[186]
Lipid nanoparticles (LNPs)	Delivery of VP28 vaccine to combat White Spot Disease (WSD) in shrimp	Superior immune protection compared to non-encapsulated vaccine	[188]
Chitosan, silver, selenium NPs	Anthelmintic effects on *Clinostomum* spp. and *Prohemistomum vivax* in *Nile tilapia* (*Oreochromis niloticus*)	All three showed insecticidal effects, with CNPs exhibiting the best performance	[194]
Ag, ZnO, Au NPs	Treatment of Ichthyophthirius multifiliis (theronts and tomonts) in rainbow trout (*Oncorhynchus mykiss*)	AgNPs (10 ng mL^−1^) caused 100% theront mortality within 2 h and inhibited tomont reproduction; reduced infectivity in vivo	[204]
Silver NPs	Treatment of *Argulus siamensis* (adult and copepodid stages) in freshwater fish (*Labeo rohita*)	100% mortality at 25–50 ppm in vitro; strong antiparasitic efficacy	[205]
Iron oxide NPs	Control of *Argulus siamensis* infestation in *Labeo rohita*	In vivo bath (2.25 mg mL^−1^, 4 days) removed 100% parasites with moderate fish safety	[206]
Chitosan–silver nanocomposites	Treatment of *Lernaea cyprinacea* infection in goldfish (*Carassius auratus*)	5.5 ppm for 24 h dislodged all parasites and promoted wound healing	[207]
Silver NPs	Anthelmintic treatment of *Cichlidogyrus* spp. (Monogenea; adults and eggs) in freshwater fish	36 µg L^−1^ for 1 h achieved 100% mortality: tegumental disruption under SEM	[208]

### 6.2. Biosensors

Biosensors are analytical devices that convert the physical or chemical signals of biomolecules into optical or electrical signals, enabling rapid, accurate, and real-time detection and quantification of biological substances [209]. By real-time monitoring of pathogens, stress biomarkers, and environmental pollutants, biosensors provide an efficient solution for the early diagnosis and integrated control of parasites [210]. The high sensitivity and specificity of biosensors greatly enhance detection efficiency. Additionally, their multifunctional capabilities not only support pathogen monitoring but also aid in assessing and optimizing the farming environment, enabling farmers to manage diseases more effectively [211].

Biosensors have achieved significant success in detecting microbial pathogens in aquaculture, particularly for common bacterial pathogens such as *Vibrio*, *Aeromonas hydrophila*, and *Flavobacterium columnare* [212]. Surface Plasmon Resonance (SPR) sensors and Optical Fiber Immunosensors (OFIs) have been successfully used to detect *Escherichia coli*, with a sensitivity of up to 94 CFU/mL [213,214]. Moreover, AuNPs can serve as key components in biosensors. In loop-mediated isothermal amplification (LAMP) technology, AuNPs enhance pathogen DNA detection efficiency by amplifying fluorescence or colorimetric signals. This method not only offers high sensitivity but also enables rapid diagnostics, and has been successfully applied for the quick detection of fish furunculosis infections [215]. Furthermore, electrochemical DNA biosensors based on AuNPs, coupled with DNA reporter probes, can be used to detect *Aphanomyces invadans* in fish. Their detection sensitivity is significantly higher than traditional PCR, with a lower detection limit of 0.5 fM for linear target DNA and 1 fM for PCR products [196].

Although most research has focused on bacteria, the potential application of biosensors in parasite treatment is also worth noting. Potentiometric biosensors have been successfully used for parasite detection [216,217]. This method combines Immunomagnetic Separation (IMS) with Enzyme-Linked Immunosorbent Assay (ELISA), enabling highly sensitive parasite diagnosis by measuring potential changes. The detection limit reaches as low as 500 cells [218,219]. The study by Santos-de-Souza et al. [220] also highlighted the potential of SPR in the rapid diagnosis of parasites. *Leishmania amazonensis*, at different stages of its lifecycle, releases an important protein marker—Cyspep (Cysteine Protease B C-terminal peptide). The study utilized Surface Plasmon Resonance (SPR) technology, where specific antibodies fixed on the sensor chip surface bind with Cyspep, to develop a sensor for real-time monitoring of this marker [220]. The method previously mentioned, combining the visual colorimetric nano-gold hybridization probe with isothermal amplification technology, can also be used for parasite detection. For example, in the case of *Toxoplasma gondii*, even as little as 1 fg of DNA in the sample can be detected [221]. Compared to traditional methods such as parasite culture and immunostaining, biosensors significantly improve detection speed and sensitivity, providing an efficient and accurate technological approach for the rapid diagnosis of parasites.

Biosensors also play a crucial role in monitoring environmental stress and fish stress biomarkers, providing significant support for the prevention and control of parasites and other diseases. Pollutants in the aquaculture environment, such as heavy metals (lead, mercury), organic compounds (phenols), as well as microplastics and nanoparticles, can accumulate and adversely affect fish health, even serving as vectors for the transmission of parasitic pathogens [210]. Biosensors based on Surface-Enhanced Raman Scattering (SERS) technology can efficiently detect the distribution and concentration of microplastics and pollutants in water, providing technical support for pollution source identification and pollution control [211]. By the real-time monitoring of water pollution levels, sensors can effectively provide early warnings of potential risks, reducing the occurrence of fish diseases caused by environmental pollution.

Fish are prone to stress under conditions such as high-density farming, temperature fluctuations, or deteriorating water quality. Stress can weaken their immune system, increasing their susceptibility to parasites and other pathogens. Cortisol, a stress hormone, is a key marker of fish stress, and its concentration changes can reflect the health status of the fish [211,222]. SPR combined with nanotechnology can sensitively detect cortisol concentrations as low as 0.001 ng/mL, enabling non-invasive real-time monitoring [223]. This provides aquaculture farmers with a quick way to assess fish health and adjust environmental conditions, such as reducing stocking density or improving water quality, thereby effectively preventing diseases triggered by stress. Although most biosensors are still in the research phase [224], with ongoing advancements in technology, they are expected to become early warning systems in aquaculture, helping farmers quickly identify potential health issues in fish and take timely intervention measures.

## 7. Conclusions

Parasite control remains a critical challenge in aquaculture. While traditional methods like chemical treatments have been effective, they are becoming less viable due to issues such as drug resistance, environmental pollution, and toxicity. As a result, new strategies are emerging, including treatments based on natural products, gene editing, and immunotherapy, as well as supportive technologies like nanotechnology and biosensors, which offer sustainable alternatives for parasite control in aquaculture.

Although novel therapeutic and auxiliary methods show potential for parasite control in aquaculture, most research still primarily focuses on bacterial pathogens, with relatively less attention given to parasites. Due to the limited knowledge of most fish parasites’ life cycles, the biological characteristics and treatment responses at different stages are not yet fully understood, making it difficult for current treatment methods to achieve comprehensive and effective control, as well as posing significant challenges in research. To overcome this challenge, future research needs to further investigate the various stages of the parasite life cycle, uncover their biological mechanisms, and provide a foundation for developing more precise and effective treatment strategies. At the same time, although emerging technologies such as gene editing and immunotherapy show great potential, their long-term effectiveness, feasibility for widespread application, and environmental impact still need further validation. Combining these new methods with environmental management measures, such as water quality control and stocking density regulation, will enhance the overall effectiveness of parasite management and drive the aquaculture industry toward more efficient and sustainable development.

## Figures and Tables

**Table 3:** Overview of Aquaculture Parasite Treatment Methods.

Category	Technique	Advantages	Disadvantages
Chemical Treatments	Traditional chemical drugs	Effective and widely available.	Resistance development, environmental toxicity.
Plant-Based Treatments	Herbal extracts	Eco-friendly and less likely to cause resistance.	Complex composition with potential side effects; inconsistent quality; narrow spectrum of efficacy; short-term therapeutic effects.
Biological Techniques	Probiotics	Eco-friendly; can be incorporated into feed for long-term prevention and treatment; enhances host immunity	Strain-specific effects; limited efficacy against certain parasites; requires prolonged use for effectiveness; sensitive to environmental conditions during storage and application
	Immunostimulants	Enhances innate immunity, suitable for young fish.	Dose-dependent effects; requires careful time control
	Vaccination	Eco-friendly and with few side effects	Difficult administration methods; limited duration of protection; challenges in commercial scalability; high complexity in development
Advanced Molecular Techniques	RNA Interference (RNAi)	Low impact on the environment and organisms	Limited duration of effectiveness; highly specific to parasite species and developmental stages; significant challenges in designing and delivering exogenous RNA
	CRISPR/Cas9 Gene Editing	Highly precise gene editing; permanent and heritable resistance	Ecological risks from escape; limited public acceptance; strict management of commercialization

## Data Availability

No new data were created or analyzed in this study. Data sharing is not applicable to this article.

## Abbreviations

The following abbreviations are used in this manuscript:

FAO	Food and Agriculture Organization
EOs	Essential oils
RNAi	RNA interference
dsRNA	Double-stranded RNA
Hsp70C	Heat shock protein 70C
iAg	Immobilized antigen
RPS	Relative protection
SOD	Superoxide dismutase
UV	Ultraviolet
NPs	Nanoparticles
MNPs	Metal Nanoparticles
CBNs	Carbon-Based Nanoparticles
LNPs	Lipid Nanoparticles
PNPs	Polymeric Nanoparticles
CNPs	Chitosan nanoparticles
PLGA	Poly (lactic-co-glycolic acid)
FDA	Food and Drug Administration
YHV	Yellow Head Virus
WSSV	White Spot Syndrome Virus
WSD	White Spot Disease
AuNPs	Gold nanoparticles
SPR	Surface Plasmon Resonance
OFIs	Optical Fiber Immunosensors
LAMP	In loop-mediated isothermal amplification
PCR	Polymerase Chain Reaction
IMS	Immunomagnetic Separation
ELISA	Enzyme-Linked Immunosorbent Assay
Cyspep	Cysteine Protease B C-terminal peptide
SERS	Surface-Enhanced Raman Scattering
